# Development and validation of the self‐stigma scale in people with diabetes

**DOI:** 10.1002/nop2.719

**Published:** 2020-12-03

**Authors:** Kawoun Seo, Youngshin Song

**Affiliations:** ^1^ Department of Nursing Joongbu University Chungnam Republic of Korea; ^2^ College of Nursing Chungnam National University Daejeon Republic of Korea

**Keywords:** diabetes mellitus, nursing, reliability, scale development, social stigma, validation

## Abstract

**Aim:**

To develop and initially validate the Diabetes Self‐Stigma Scale for assessing self‐stigma in people with diabetes.

**Design:**

Scale development and evaluation.

**Methods:**

Participants were 399 patients with diabetes. In phase 1, initial items were generated based on the concept analysis of diabetes self‐stigma. Moreover, content validity was established by diabetes experts. Phase 2 evaluated structural validity through item analysis, exploratory factor analysis and confirmatory factor analysis. Reliability was evaluated by examining stability and internal consistency.

**Results:**

The findings revealed that the self‐stigma scale for patients with diabetes is a valid and reliable instrument. The Diabetes Self‐Stigma Scale was confirmed with 16 items. It consists of four domains: comparative inability, social withdrawal, self‐devaluation and apprehensive feeling. The scale developed in this study can measure self‐stigma in diabetes patients and can be used as an intervention to reduce self‐stigma.

## 1 INTRODUCTION

Diabetes is one of the most common lifestyle diseases in the world, and it is necessary for people with diabetes to maintain adequate blood glucose levels. Failure to maintain adequate blood sugar levels can result in exposure to several complications (International Diabetes Foundation, 2017), reducing their quality of life (Shao et al., 2019). In particular, due to lack of insulin in the body, insulin treatment is absolutely necessary for type 1 diabetes, whereas type 2 diabetes patients can control blood sugar through diet or exercise, so self‐management is especially important for them (Ahola & Groop, 2013; Kim et al., 2017). Self‐stigma means that individuals accept socially shared stereotypes and prejudices, feel that they are socially unacceptable and devalue themselves. Self‐stigma reduces self‐esteem, self‐efficacy and overall quality of life (Corrigan et al., 2006, 2009; Vass et al., 2017). In addition, high levels of self‐stigmatization in people with chronic conditions such as diabetes may negatively affect self‐management if such individuals avoid treatment or reduce compliance (Kato et al., 2016; Link et al., 2001; Sirey et al., 2001). Therefore, there is a need to improve this situation by measuring the degree of self‐stigma in people with diabetes.

Stigma for diabetes has already been discussed in several studies (Browne et al., 2016; Kato et al., 2014, 2016; Song & Ah, 2016). However, previous studies do not clearly focus on the types of stigma of diabetes, but simply refer to it as “stigma” or “social stigma” (Seo & Song, 2019). Patients with diabetes can hide their disease because their symptoms are not visible (Song & Ah, 2016); thus, self‐stigma, which is related to self‐perception, is more important than social stigma.

In Japan, the validity and reliability of related tools to measure self‐stigma for diabetes have been established (Kato et al., 2014). However, since the Japanese version of the Self‐Stigma Scale was initially developed for minorities in Hong Kong (Mak & Cheung, 2010), it does not fully reflect the characteristics of people with diabetes who experience self‐stigma. In addition, self‐stigma is created by the internalization of social stigma and social stigma is influenced by the culture to which they belong (Rüsch et al., 2005; Seo & Song, 2019). Therefore, there is a limit to its usefulness in measuring self‐stigma in Korean people with diabetes. Despite the need to measure and manage self‐stigma of diabetes patients, there are no tools to measure self‐stigma for diabetes patient in Korea. Therefore, it is necessary to develop and validate the Diabetes Self‐Stigma Scale (DSSS) for people with diabetes in Korea that accurately reflects the properties of self‐stigma.

## 2 BACKGROUND

It has been found that stigma reduces self‐esteem (Park et al., 2019; Rüsch et al., 2005). However, not all social stereotypes or prejudices reduce an individual's self‐esteem. To do this, the stereotypes or prejudices of society must be internalized. Such internalization is called self‐stigma. Self‐stigma means that individuals accept socially shared stereotypes and prejudices and devalue themselves, believing that they are merely socially tolerated (Vogel et al., 2006). Self‐stigma occurs in three stages: awareness, agreement and application (Corrigan, 2004; Corrigan & Watson, 2002). This can be explained by an individual identifying one's own traits with those of the stigmatized group and applying stereotypes and prejudices related to them (Eisenberg et al., 2009). Individuals who experience social stigma may be aware of stereotypes that others apply to them, but they do not internalize all of them. In other words, self‐stigma is not simply the awareness of prejudice or stereotype but the process of internalization.

A previous study (Link et al., 1989) emphasized the importance of self‐stigma by suggesting a “modified labelling theory.” This means that people with self‐stigma experience negative consequences, such as damage to their self‐esteem, that make them vulnerable. Therefore, individuals with self‐stigma have a diminished self‐esteem or self‐worth (Vogel et al., 2006). They perceive themselves as inferior, inadequate or weak (Nadler & Jeffrey, 1986).

In this context, people with diabetes often develop self‐stigma through the treatment process rather than because of the symptoms of the disease. Self‐stigma is more important than social stigma for diabetes because the general public does not think that having diabetes is stigmatized and people with diabetes do not stigmatize other diabetes patients. In contrast, diabetes patients develop negative attitudes towards themselves because they feel judged and monitored by others (Schabert et al., 2013). In addition, patients with diabetes can hide their disease from others because their disease is not visible (Song & Ah, 2016).

Most studies on stigma of diabetes patients have been conducted to define the concept simply as stigma or social stigma without considering its level (Abdoli et al., 2018; Browne et al., 2016; Schabert et al., 2013). Nishio and Chujo (2017) conducted a study on self‐stigma in diabetes patients that was limited to patients with type 1 diabetes and may not be generalizable to patients with type 2 diabetes. Additionally, the tool that was designed to measure diabetes self‐stigma is the only DSSS used in Japan (Kato et al., 2014). This tool was designed for minorities with mental illness and homosexuals in Hong Kong at the time of the tool's development. It is therefore limited in that it does not sufficiently reflect the characteristics of diabetes self‐stigma. In addition, since self‐stigma affects public perception about the disease (Kato et al., 2014), it is necessary to have a self‐stigma tool that applies to Korean culture.

## 3 THE STUDY

### 3.1 Design

The design of this study involved a development phase and an evaluation phase of the scale. The participants were patients with diabetes recruited from the outpatient endocrine departments at two university hospitals in D city and from two health centres located in cities C and S. A sample of 399 people was included in the study. The study was explained to them; they agreed to participate and provided written consent. The sample was randomly divided into set A for exploratory factor analysis (EFA) and set B for confirmatory factor analysis (CFA).

The inclusion criteria for participants were as follows: (a) received a diabetes diagnosis from a physician, (b) participated in functional self‐care and possible social activities, (c) were not diagnosed with psychiatric illnesses and were able to communicate, (d) had the cognitive ability to express feelings and (e) were voluntarily participating in the study.

**FIGURE 1:**
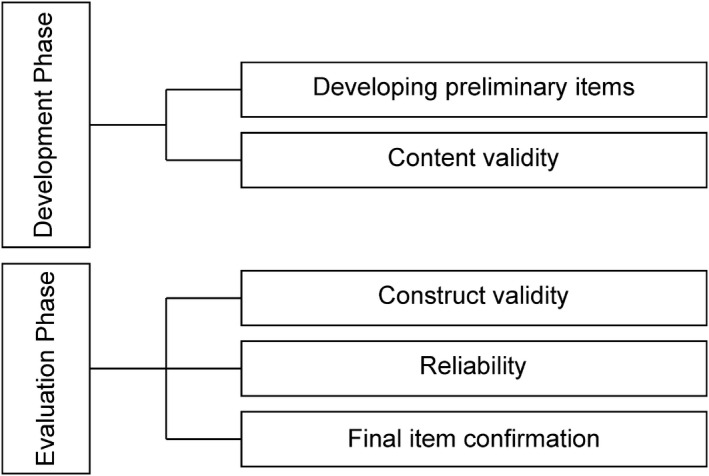
Development process of the self‐stigma scale for people with diabetes mellitus

This study was conducted in two phases (Figure 1) (Netemeyer et al., 2003). In the development phase, preliminary questions were developed based on the definitions and attributes of the concepts presented through concept analysis (Seo & Song, 2019). The preliminary tool underwent content validation with the expert group and diabetes patients group. In the evaluation phase, structural validity was evaluated through EFA and CFA. In addition, the final instrument of diabetes self‐stigma was confirmed by evaluating reliability using internal consistency.

### 3.2 Method

#### 3.2.1 Development phase

The initial items of the DSSS were developed based on the results of concept analysis of diabetes self‐stigma to improve the availability of the contents. The concept analysis for self‐stigma was conducted through three phases (theoretical phase, fieldwork phase and final analysis phase) of a hybrid method for Korean individuals with diabetes, reflecting the self‐stigma of diabetes based on Korean culture. The self‐stigma concept included three categories with nine attributes: affective (negative feelings and feeling sorry for others who have concerns about me), cognitive (low self‐esteem and self‐ efficacy, perceived weakness, low expectations for the future, worry for children and disease burden) and behavioural (social withdrawal and avoiding disease disclosure) factors. Detailed methods and results can be found in our main published paper (Seo & Song, 2019). Based on this, 40 questions were derived using existing self‐stigma tools and in‐depth interview data of patients with diabetes.

#### 3.2.2 Evaluation phase

In this phase, the content validity index (CVI) was calculated. The content was validated with experts and people with diabetes. Ten experts participated including endocrinologists, nurses specializing in diabetes and nursing professors. Ten diabetes patients also participated in the content verification. They were asked to respond on a 4‐point Likert scale as to whether the question was appropriate for measuring diabetes self‐stigma. In addition, they were required to pick out words that were used inappropriately or sentences that were difficult to understand. CVI measures the number of experts who gave a 3 or 4 rating for each item after measuring “1 = *not relevant*,” “2 = *somewhat relevant*,” “3 = *relevant*” and “4 = *very relevant*.” That is, the proportion in agreement about the relevance was measured and an item with a score of 0.8 or higher was adopted for the scale. Items with scores below 0.5 were considered less valid and were excluded from the list by obtaining a non‐consensus ratio of relevance and items with values of 0.5–0.8 were revised based on the opinions of experts (Polit et al., 2007).

Structural validity was confirmed through item analysis, EFA and CFA. The EFA determines the number of dimensions by grouping related questions. CFA is a method of verifying support by modelling theoretically proven dimensions of self‐stigma for diabetes patients and items tied to EFA (Kang, 2006). In addition, the final questions were confirmed after evaluating their reliability using internal consistency.

#### 3.2.3 Participants

This study was conducted with adults over 20 years of age with type 2 diabetes. Convenience sampling was conducted for those who understood the purpose of the study and voluntarily agreed to participate in the study among patients with diabetes who visited the University Hospital Endocrinology Department in D City and the public health centre in C Province. Data were collected from a total of 430 subjects; this number was reached based on the evidence that 5–10 times (or more) the number of items was appropriate for a stable test of the reliability and validity of the measurement tool including factor analysis (Tabachnick et al., 2001). Of these, data were analysed using the survey results of 399 people, excluding the data from 31 people with insufficient responses. Of the 399 responses, data from 183 people (Dataset A) were used for exploratory factor analysis and the data from 216 people (Dataset B) were used for confirmatory factor analysis.

The administered questionnaire included sociodemographic information (i.e. gender, age, educational level, marital status, having religion, having a job, cohabiting status, economic satisfaction, social activities, perceived health status) and characteristics of diabetes (i.e. duration of illness, type of medical care, how diabetes is managed, number and type of complications, receiving diabetes education).

### 3.3 Statistical analysis

The developed tool was analysed using IBM SPSS (version 22.0) and AMOS (version 22.0). Item‐total correlation coefficients were calculated to determine whether each item reflects the concept of self‐stigma measurement. Items with low contributions in the domain with the item‐total score correlation coefficient of less than 0.20 were deleted (Field, 2005).

The validity of this tool was analysed using EFA and CFA. EFA examined the suitability of the factor analysis using the Kaiser–Meyer–Olkin (KMO) index and Bartlett's spherical test. EFA was then conducted to determine the number of dimensions by grouping related items. To conduct EFA, it was necessary to determine the factor extraction and rotation methods. Factor extraction includes principal component analysis, principal axis factor method and maximum likelihood method. Among them, the principal factor method assumes a factor analysis model and extracts factors so that the covariance of the measured variables can be explained as much as possible. Rotation was then performed to simplify the structure of the factors. Both orthogonal and oblique rotation methods were used. Oblique rotation is more common in the field of social science because it places a greater significance on the interpretation of the factor structure in terms of the disconnection of factors (Woo, 2012). In this study, factors were extracted using the principal factor method. Thereafter, the oblimin method, which is an orthogonal pre‐transaction method, was used. Standards for loading values vary between 0.3, 0.4 and 0.5, but in this study, items with communality and loading value size of 0.4 or less were removed (Song, 2011).

In CFA, indices that explain the adequacy of the model include chi‐squared to degree of freedom ratio (CMIN/*df*), root mean square residual (RMR), root mean square error of approximation (RMSEA), goodness‐of‐fit index (GFI), adjusted goodness‐of‐fit index (AGFI) and comparative fitness index (CFI). When CMIN/*df* < 2.0, RMR < 0.08, RMSEA < 0.05, GFI > 0.9, AGFI > 0.9, or CFI > 0.9, the test model is judged to be suitable. The significance of the factor load of each item was verified to confirm the concentration validity.

The reliability of this tool was analysed using Cronbach's α, which was also used to identify the internal consistency. If the Cronbach's α value is 0.7–0.8, internal consistency is good; if it is 0.8–0.9, it can be regarded as very high. However, if it is 0.9 or higher, the number of questions should be reduced (DeVellis, 2016).

### 3.4 Ethics

The study was approved by the Institutional Review Board of the nursing college of [blinded for peer review] (No. IRB 2‐1046881‐A‐N‐01‐201705‐HR‐012). When recruiting participants, the purpose of the study was explained to them and data were collected only from those who had understood and agreed in writing. Participants could withdraw from the study at any time, if desired, with no consequence. Participants were informed that all data collected during the study would be used for research purposes and discarded two years after the completed study. They were also informed that the results could be shared if requested by the government or a review agency. All participants were provided with incentives such as an eco‐bag and toothbrush.

## 4 RESULTS

### 4.1 Participants’ characteristics

Table 1 shows the general and disease‐related characteristics of 399 participants. There were 42.9% and 57.1% male and female participants, respectively. Their average age was 65.88 years. The mean duration of diabetes was 11.52 years, and the most common cases were managed by university hospitals (45.9%). Of the participants, 74.4% managed their blood sugar with oral medication and only 6.8% used insulin therapy. The data were divided into two sets for EFA and CFA analysis through randomization.

**TABLE 1:** General and disease‐related characteristics of the participants (*N* = 399)

Characteristics	Categories	Total	Data A (*n* = 183)	Data B (*n* = 216)	χ^2^ or *t*	*p*
		*N* (%) or *M* (±*SD*)				
Gender	Male	171 (42.9)	72 (39.3)	99 (45.8)	1.70	.223
	Female	228 (57.1)	111 (60.7)	117 (54.2)		
Age (years)		65.88 (±11.8)	67.19 (±12.2)	65.07 (±11.4)	1.78	.075
	≤49	33 (8.3)	14 (7.7)	19 (8.8)		
	50–59	84 (21.1)	33 (18.0)	51 (23.6)		
	60–69	113 (28.3)	51 (27.9)	62 (28.7)		
	≥70	169 (42.3)	85 (46.4)	84 (38.9)		
Education level	Uneducated	47 (11.8)	30 (16.4)	37 (17.1)	7.61	.107
	Elementary school	88 (22.0)	51 (27.9)	37 (17.1)		
	Middle school	63 (15.8)	27 (14.7)	36 (16.7)		
	High school	120 (30.1)	47 (25.7)	73 (33.8)		
	College	81 (20.3)	28 (15.3)	33 (15.3)		
Marital status	Yes	293 (73.4)	132 (72.1)	161 (74.5)	1.11	.573
	No	106 (26.6)	51 (27.9)	55 (25.5)		
Having religion	Yes	226 (56.6)	104 (56.8)	122 (56.5)	0.00	1.00
	No	173 (43.4)	79 (43.2)	94 (43.5)		
Having a job	Yes	163 (40.9)	73 (39.9)	90 (41.7)	0.12	.760
	No	233 (59.1)	110 (60.1)	126 (58.3)		
Cohabiting status	Solitary	72 (18.0)	34 (18.5)	38 (17.6)	3.12	.373
	With spouse	211 (52.9)	92 (50.3)	119 (55.1)		
	With offspring	101 (25.3)	47 (25.7)	54 (25.0)		
	Other	15 (3.8)	10 (5.5)	5 (2.3)		
Economic satisfaction	Very good	6 (1.5)	1 (0.5)	5 (2.3)	3.32	.506
	Good	99 (24.8)	46 (25.1)	53 (24.5)		
	Fair	255 (63.9)	116 (63.4)	139 (64.4)		
	Poor	31 (7.8)	17 (9.3)	14 (6.5)		
	Very poor	8 (2.0)	3 (1.7)	5 (2.3)		
Social activities	Everyday	38 (9.5)	22 (12.0)	16 (7.4)	6.01	.198
	2–3 times per week	82 (20.6)	35 (19.1)	47 (21.8)		
	1 time per week	59 (14.8)	24 (13.1)	35 (16.2)		
	2–3 times per month	93 (23.3)	37 (20.2)	65 (25.9)		
	1 time per month	127 (31.8)	65 (35.6)	62 (28.7)		
Perceived health status	Very good	2 (0.5)	0 (0.0)	2 (1.0)	3.26	.515
	Good	78 (19.5)	38 (20.8)	40 (18.5)		
	Fair	182 (45.6)	78 (42.6)	104 (48.1)		
	Poor	124 (31.1)	60 (32.8)	64 (29.6)		
	Very poor	13 (3.3)	7 (3.8)	6 (2.8)		
Duration of diabetes (years)		11.52 (±9.40)	12.25 (± 9.7)	11.21 (± 9.2)	1.09	.275
	≤5	138 (34.6)	61 (33.3)	77 (35.6)		
	6–10	109 (27.3)	49 (26.8)	60 (27.8)		
	11–20	98 (24.6)	43 (23.5)	55 (25.5)		
	≥21	54 (13.5)	30 (16.4)	24 (11.1)		
Type of hospital	Clinic	94 (23.5)	48 (26.2)	46 (21.3)	3.41	.332
	General hospital	79 (19.8)	36 (19.7)	43 (19.9)		
	University hospital	183 (45.9)	76 (41.5)	107 (49.5)		
	Public health centre	43 (10.8)	23 (12.6)	20 (9.3)		
Type of medication	PO	297 (74.4)	135 (73.8)	162 (75.0)	2.78	.427
	PO + insulin	63 (15.8)	16 (8.7)	11 (5.1)		
	Insulin	27 (6.8)	28 (15.3)	35 (16.2)		
	Diet therapy	12 (3.0)	4 (2.2)	8 (3.7)		
Experience of diabetes education	Yes	229 (57.4)	100 (54.6)	129 (59.7)	1.04	.312
	No	170 (42.6)	83 (45.4)	87 (40.3)		
Having complications	Yes	99 (24.8)	53 (29.0)	47 (21.8)	2.73	.106
	No	300 (75.2)	130 (71.0)	169 (78.2)		
Type of complication (*n* = 89)	Cardiovascular disease	45 (11.3)	23 (12.6)	22 (10.2)	3.22	.666
	Kidney disease	16 (4.0)	12 (6.6)	4 (1.9)		
	Cerebrovascular disease	11 (2.8)	6 (3.3)	5 (2.3)		
	Neurological disease	10 (2.5)	6 (3.3)	4 (1.9)		
	Ocular disease	45 (11.3)	23 (12.6)	22 (10.2)		
	Foot disease	4 (1.0)	2 (1.1)	2 (0.9)		

### 4.2 Development phase

The initial question of the DSSS was developed based on the definition, dimensions and attributes obtained during concept analysis of self‐stigma for diabetes (Seo & Song, 2019). Results of concept analysis revealed that the scale was composed of nine attributes in three dimensions: affective, cognitive and behavioural factors. The nine attributes were divided into 23 indicators; using these indicators and in‐depth interview data from patients with diabetes, the first 40 questions were produced. The response format involved a five‐point Likert scale to continuously measure the opinions, attitudes and characteristics of the respondents.

### 4.3 Evaluation phase

#### 4.3.1 Content validity

After content validity, the 8th and 12th items, which had CVI lower than 0.5, were eliminated. Moreover, the 14^th^, 22^nd^, 26^th^, 29^th^, 31^st^ and 35^th^ items were incorporated into similar items. In addition, the 17th item, which the patients considered irritating, was deleted. Additionally, the 6^th^, 20^th^, 24^th^, 28^th^ and 32^nd^ items were deleted. Thus, altogether, 14 items were eliminated, and one item was added according to expert opinion; the original 40 items were reduced to 27 items.

#### 4.3.2 Item analysis

Based on the result of the item analysis, two items (37^th^ & 38^th^) with a total correlation coefficient of less than 0.20 were deleted. The resulting scale consisted of 25 items. The reliability of the tool increased from 0.902–0.909.

#### 4.3.3 Exploratory factor analysis

In the first sample (*n* = 183), the KMO value for the EFA of 25 items was 0.873 and the Bartlett's spherical test was 0.001 (χ^2^ = 2,104.62, *p* < .001). When the common extraction values of the variables were analysed using the principal component analysis, the commonalities of four items were less than 0.4 and they were excluded. The four factors extracted were the following: (a) comparative inability, (b) social withdrawal, (c) self‐devaluation and (d) apprehensive feeling, as shown in Table 2.

**TABLE 2:** Item loadings from rotated component matrix

Item	Communalities	Factor 1 Comprehensive inability	Factor 2 Social withdrawal	Factor 3 Self‐devaluation	Factor 4 Apprehensive feeling
T 18	0.644	**0.746**	0.142	0.191	0.173
T 16	0.751	**0.668**	0.440	0.320	0.092
T 15	0.657	**0.655**	0.397	0.221	0.147
T 19	0.454	**0.643**	0.080	0.086	0.163
T 13	0.560	**0.606**	0.290	0.169	0.282
T 25	0.318	**0.450**	−0.039	0.102	0.322
T 5	0.381	**0.425**	0.100	0.237	0.366
T 34	0.738	0.211	**0.812**	0.159	0.099
T 36	0.591	0.013	**0.697**	0.272	0.176
T 33	0.561	0.103	**0.664**	0.310	0.114
T 39	0.272	0.186	**0.448**	0.039	0.187
T 30	0.466	0.304	**0.421**	0.343	0.281
T 40	0.326	0.262	**0.388**	−0.022	0.325
T 9	0.680	0.136	0.101	**0.794**	0.144
T 10	0.543	0.185	0.171	**0.692**	−0.018
T 7	0.427	0.177	0.286	**0.536**	0.161
T 11	0.516	0.367	0.296	**0.483**	0.245
T 41	0.578	0.157	0.188	0.236	**0.680**
T 4	0.442	0.108	0.094	0.064	**0.646**
T 2	0.530	0.332	0.186	0.033	**0.620**
T 1	0.314	0.161	0.189	0.061	**0.498**
Eigen value		7.31	1.43	1.09	0.90
Variance, %		34.83	6.82	5.19	4.32
Cumulative variance, %		34.83	41.65	46.85	51.17

Note

Extraction method was principal axis analysis with varimax rotation.

The bold values represent the final selected factor loading values

#### 4.3.4 Confirmatory factorial analysis

**FIGURE 2:**
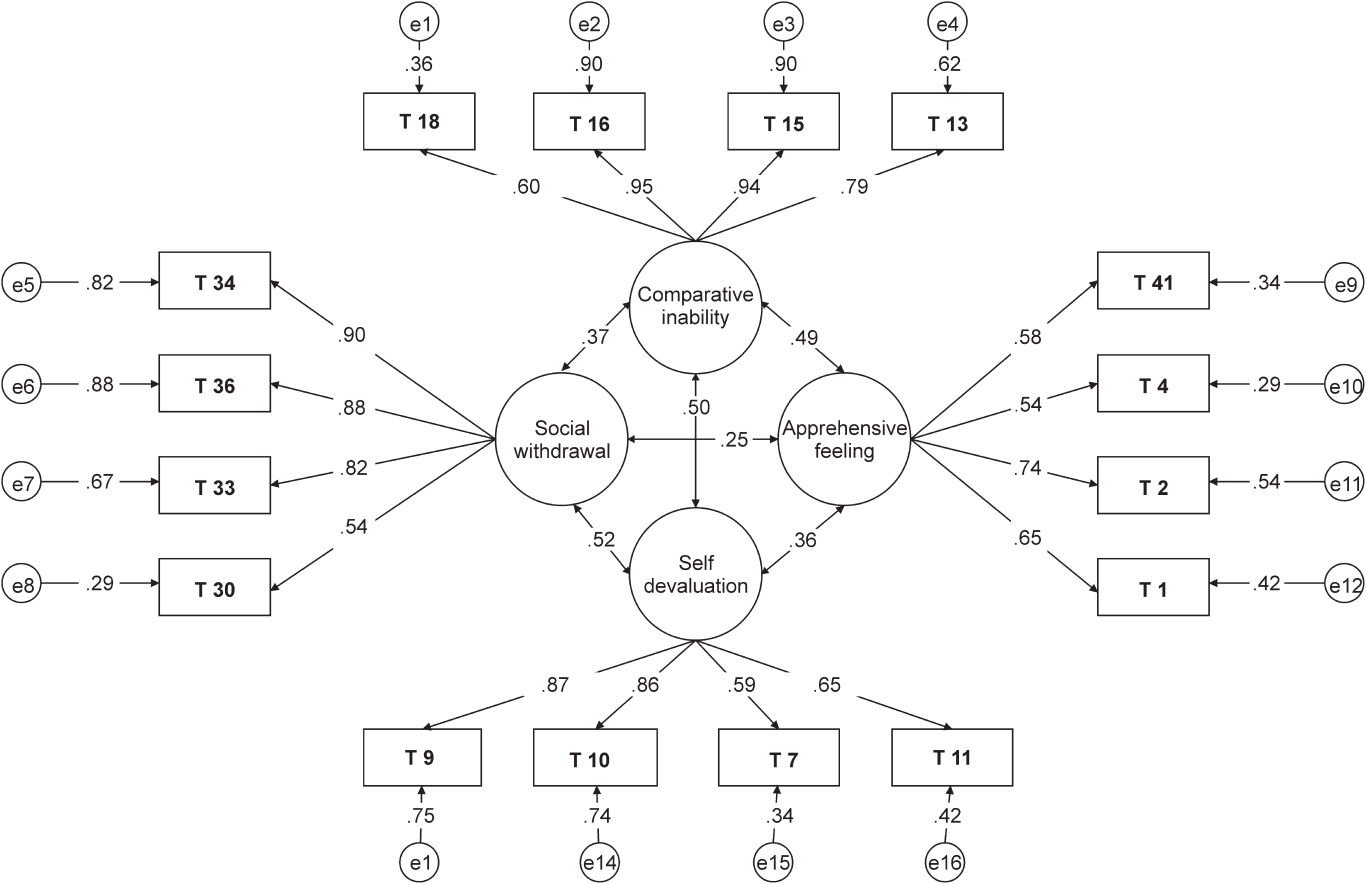
The self‐stigma model for patients with diabetes mellitus

In the second sample (*N* = 216), five items were deleted and the model's goodness‐of‐fit test was performed according to the criteria that the factor loading should not be less than 0.40 or more than 0.95 (Song, 2011). When analysing 16 items of the four factors that were divided into comparative inability, social withdrawal, self‐devaluation and apprehensive feeling, the chi‐squared degree of freedom (χ^2^/*df*) was 2.742. The result of the model's goodness of fit is shown in Table 3. Models were found to be suitable for CFI and RMR, but GFI, AGFI and RMSEA were found to be inadequate. Standardized factor loadings ranged from 0.54–0.95 (*p* < .001) (Figure 2).

**TABLE 3:** Goodness‐of‐fit indicators of the confirmatory factor analysis

Model fit statistics	χ^2^	*p*	Absolute fit index			Incremental fit index		
			CMIN/DF	SRMR	RMSEA	GFI	AGFI	CFI
Model	268.71	<.001	2.742	0.149	0.090	0.867	0.816	0.910
Standard		(<.001)	<3.0	≤0.05 or ≤0.10	≤0.05 or ≤0.10	>0.9	>0.9	>0.9

Abbreviations: SRMR, standardized root mean square residual; RMSEA, root mean square error of approximation; GFI, goodness‐of‐fit index; AGFI, adjusted goodness‐of‐fit index; CFI, comparative fit index.

### 4.4 Reliability

Cronbach's coefficient of the developed tool was 0.89. Cronbach's values for each factor are as follows: comparative inability 0.88, social withdrawal 0.84, self‐devaluation 0.80 and apprehensive feeling 0.74. The tool showed good internal consistency, indicating that it was reliable.

## 5 DISCUSSION

The purpose of this study was to develop and test a measure of self‐stigma, which negatively affects the psychological well‐being of patients with diabetes. This tool was developed based on the results of the concept analysis of diabetes self‐stigma in Korea. The result of EFA shows four factors, namely comparative inability, social withdrawal, self‐devaluation and apprehensive feeling, which are unlike the three‐dimensional structure (cognitive, behavioural and emotional factors) in the concept analysis of diabetes self‐stigma (Seo & Song, 2019). This is in line with Corrigan et al. (2012), who described the process of self‐stigmatization according to the stages of awareness, agreement, application and harm to self‐esteem. Awareness is a perception of social stigma and a cognitive aspect of self‐stigma. It is a comparative inability factor, suggesting that a role cannot be performed due to a lack of health. Agreement correlates with the factor of self‐devaluation, as it indicates that a person identifies with social stigma. Application refers to the behavioural aspect of self‐stigma and is consistent with the social withdrawal factor, which includes difficulty in or discomfort pertaining to attending meetings due to diabetes. Harm to self‐esteem can be explained by the emotional aspects that result from such cognition and behaviour, consistent with factors of the apprehensive feeling that include embarrassment, resentment and fear caused by diabetes (Corrigan et al., 2012). Therefore, the DSSS covers both the formation and the consequent aspects of self‐stigma.

For refining the items, the content validity was examined with diabetes specialists and patients with diabetes, the target group of the tool. In this process, some participants wept in response to questions. The self‐stigma scores of the participants who wept were high. They were likely to have encountered many negative factors during self‐care while managing diabetes and psychological damage and/or stress may have been high in the process. In fact, diabetes patients complained of emotional distress due to their lifestyle and restrictions and they found it difficult to live up to the recommendations due to limited guidelines (Aljuaid et al., 2018). However, because of insufficient research on these areas in Korea, qualitative studies on the psychological state of people with diabetes and active efforts are needed to solve this problem.

The tool was confirmed with the final 16 items. This is similar to the 12‐item Weight Self‐Stigma Scale (WSSS) (Lillis et al., 2010) and the 16‐item The Self‐Stigma of Depression Scale (SSDS) (Barney et al., 2010). The explanatory power of the confirmed scale was 51%, and the reliability had a Cronbach's α value of 0.89, indicating high internal consistency. This is slightly higher than the 0.878 (Lillis et al., 2010) of WSSS and the 0.87 (Barney et al., 2010) of the SSDS. However, it is lower than the 0.96 of the Japanese version of the Self‐Stigma Scale (Kato et al., 2014). On the other hand, compared with the Japanese version, the shorter and simpler questions in this version can be more easily answered. In addition, since the scale was constructed based on the concept analysis of diabetes, it has the advantage of sufficiently reflecting the characteristics of diabetes.

### 5.1 Limitations

CFI was supported by CFA to confirm the theoretical structure of self‐stigma, but there is a limitation in terms of the low value of CFI and AGFI. It can be inferred that the reason for this was the sampling from a single region. Woo (2012) reported that the model fit is low when measurement factors that differ from each other are inserted simultaneously. Because DSSS was developed based on the Korean culture, there are limitations to the generalizability. That is, there is a limitation to measuring and comparing self‐stigma between cultures using DSSS. Therefore, it is necessary to verify the cross‐cultural validity through a large‐sample survey in further studies.

## 6 CONCLUSION

Based on Corrigan's self‐stigma perspective, this study developed a 16‐item DSSS that can measure the multidimensional concept of self‐stigma for diabetes patients. The DSSS has secured practicality by refining items, including by establishing validity and reliability. This can contribute to the development of a nursing theory of self‐stigma for diabetes. It is expected that the self‐stigma measurement of patients with diabetes through the DSSS will help nurses comprehensively understand their patients and provide a basis for differentiated diabetes management strategies according to the degree of self‐stigma. In addition, as a strategy to alleviate diabetes self‐stigma, the constitutive factors derived through this study can be used as indices constituting the sub‐domains in providing nursing interventions for self‐stigma.

## CONFLICT OF INTEREST

No conflict of interest has been declared by the authors.

## Data Availability

All data generated during this study are included in this published article.

## References

[nop2719-bib-0001] Abdoli, S. , Doosti, I. M. , Hardy, L. R. , & Funnell, M. (2018). A discussion paper on stigmatizing features of diabetes. Nursing Open, 5(2), 113–119. 10.1002/nop2.112 29599986 PMC5867293

[nop2719-bib-0002] Ahola, A. , & Groop, P. H. (2013). Barriers to self‐management of diabetes. Diabetic Medicine, 30(4), 413–420. 10.1111/dme.12105 23278342

[nop2719-bib-0003] Aljuaid, M. O. , Almutairi, A. M. , Assiri, M. A. , Almalki, D. M. , & Alswat, K. (2018). Diabetes‐related distress assessment among type 2 diabetes patients. Journal of Diabetes Research, 2018, 1–10. 10.1155/2018/7328128 PMC5892264 29770340

[nop2719-bib-0004] Barney, L. J. , Griffiths, K. M. , Christensen, H. , & Jorm, A. F. (2010). The Self‐Stigma of Depression Scale (SSDS): Development and psychometric evaluation of a new instrument. International Journal of Methods in Psychiatric Research, 19(4), 243–254. 10.1002/mpr.325 20683846 PMC6878480

[nop2719-bib-0005] Browne, J. L. , Ventura, A. D. , Mosely, K. , & Speight, J. (2016). Measuring the stigma surrounding type 2 diabetes: Development and validation of the Type 2 Diabetes Stigma Assessment Scale (DSAS‐2). Diabetes Care, 39(12), 2141–2148. 10.2337/dc16-0117 27515964

[nop2719-bib-0006] Corrigan, P. (2004). How stigma interferes with mental health care. American Psychologist, 59(7), 614. 10.1037/0003-066X.59.7.614 15491256

[nop2719-bib-0007] Corrigan, P. W. , Larson, J. E. , & Rüsch, N. (2009). Self‐stigma and the “why try” effect: Impact on life goals and evidence‐based practices. World Psychiatry, 8(2), 75–81. 10.1002/j.2051-5545.2009.tb00218.x 19516923 PMC2694098

[nop2719-bib-0008] Corrigan, P. W. , Michaels, P. J. , Vega, E. , Gause, M. , Watson, A. C. , & Rusch, N. (2012). Self‐stigma of mental illness scale‐short form: Reliability and validity. Psychiatry Research, 199(1), 65–69. 10.1016/j.psychres.2012.04.009 22578819 PMC3439592

[nop2719-bib-0009] Corrigan, P. W. , & Watson, A. C. (2002). The paradox of self‐stigma and mental illness. Clinical Psychology: Science and Practice, 9, 35–53. 10.1093/clipsy.9.1.35

[nop2719-bib-0010] Corrigan, P. W. , Watson, A. C. , & Barr, L. (2006). The self‐stigma of mental illness: Implications for self‐esteem and self‐efficacy. Journal of Social and Clinical Psychology, 25(8), 875–884. 10.1521/jscp.2006.25.8.875

[nop2719-bib-0011] DeVellis, R. F. (2016). Scale development: Theory and applications (Vol. 26). Sage Publications.

[nop2719-bib-0012] Eisenberg, D. , Downs, M. F. , Golberstein, E. , & Zivin, K. (2009). Stigma and help seeking for mental health among college students. Medical Care Research and Review, 66(5), 522–541. 10.1177/1077558709335173 19454625

[nop2719-bib-0555] Field, A. (2005). Exploratory factor analysis. Discovering statistics using SPSS. 619–680.

[nop2719-bib-0013] International Diabetes Foundation (2017). International Diabetes Federation diabetes atlas (8th ed.). International Diabetes Foundation.

[nop2719-bib-0014] Kang, Y. A. (2006). Personal knowledge and caring. Humanity Journal, 20(1), 117–144.

[nop2719-bib-0015] Kato, A. , Fujimaki, Y. , Fujimori, S. , Isogawa, A. , Onishi, Y. , Suzuki, R. , Yamauchi, T. , Ueki, K. , Kadowaki, T. , & Hashimoto, H. (2016). Association between self‐stigma and self‐care behaviors in patients with type 2 diabetes: A cross‐sectional study. BMJ Open Diabetes Research & Care, 4(1), e000156. 10.1136/bmjdrc-2015-000156 PMC4716123 26835138

[nop2719-bib-0016] Kato, A. , Takada, M. , & Hashimoto, H. (2014). Reliability and validity of the Japanese version of the Self‐Stigma Scale in patients with type 2 diabetes. Health and Quality of Life Outcomes, 12, 179. 10.1186/s12955-014-0179-z 25495723 PMC4297463

[nop2719-bib-0017] Kim, J. H. , Kim, K. Y. , Lee, S. J. , Bae, S. G. , Ryu, D. H. , & Lee, W. K. (2017). A study on sustainability of knowledge, self‐care behavior and self‐efficacy after self‐help group program for hypertension and diabetes. Journal of Health Informatics and Statistics, 42(3), 276–284. 10.21032/jhis.2017.42.3.276

[nop2719-bib-0018] Lillis, J. , Luoma, J. B. , Levin, M. E. , & Hayes, S. C. (2010). Measuring weight self‐stigma: The weight self‐stigma questionnaire. Obesity, 18(5), 971–976. 10.1038/oby.2009.353 19834462

[nop2719-bib-0019] Link, B. G. , Cullen, F. T. , Struening, E. , Shrout, P. E. , & Dohrenwend, B. P. (1989). A modified labeling theory approach to mental disorders: An empirical assessment. American Sociological Review, 54(3), 400–423. 10.2307/2095613

[nop2719-bib-0020] Link, B. G. , Struening, E. L. , Neese‐Todd, S. , Asmussen, S. , & Phelan, J. C. (2001). Stigma as a barrier to recovery: The consequences of stigma for the self‐esteem of people with mental illnesses. Psychiatric Services, 52(12), 1621–1626. 10.1176/appi.ps.52.12.1621 11726753

[nop2719-bib-0021] Mak, W. W. , & Cheung, R. Y. (2010). Self‐stigma among concealable minorities in Hong Kong: Conceptualization and unified measurement. American Journal of Orthopsychiatry, 80(2), 267–281. 10.1111/j.1939-0025.2010.01030.x 20553520

[nop2719-bib-0022] Nadler, A. , & Jeffrey, D. (1986). The role of threat to self‐esteem and perceived control in recipient reaction to help: Theory development and empirical validation. Advances in Experimental Social Psychology, 19, 81–122. 10.1016/S0065-2601(08)60213-0

[nop2719-bib-0023] Netemeyer, R. G. , Bearden, W. O. , & Sharma, S. (2003). Scaling procedures: Issues and applications. Sage Publications. 10.4135/9781412985772

[nop2719-bib-0024] Nishio, I. , & Chujo, M. (2017). Self‐stigma of patients with type 1 diabetes and their coping strategies. Yonago Acta Medica, 60(3), 167–173. 10.33160/yam.2017.09.005 28959127 PMC5611471

[nop2719-bib-0025] Park, K. , Lee, M. H. L. , & Seo, M. (2019). The impact of self‐stigma on self‐esteem among persons with different mental disorders. International Journal of Social Psychiatry, 65(7–8), 558–565. 10.1177/0020764019867352 31373252

[nop2719-bib-0026] Polit, D. F. , Beck, C. T. , & Owen, S. V. (2007). Is the CVI an acceptable indicator of content validity? Appraisal and recommendations. Research in Nursing & Health, 30(4), 459–467. 10.1002/nur.20199 17654487

[nop2719-bib-0027] Rüsch, N. , Angermeyer, M. C. , & Corrigan, P. W. (2005). Mental illness stigma: Concepts, consequences and initiatives to reduce stigma. European Psychiatry, 20(8), 529–539. 10.1016/j.eurpsy.2005.04.004 16171984

[nop2719-bib-0028] Schabert, J. , Browne, J. L. , Mosely, K. , & Speight, J. (2013). Social stigma in diabetes: A framework to understand a growing problem for an increasing epidemic. The Patient – Patient‐Centered Outcomes Research, 6(1), 1–10. 10.1007/s40271-012-0001-0 23322536

[nop2719-bib-0029] Seo, K. , & Song, Y. (2019). Self‐stigma among Korean patients with diabetes: A concept analysis. Journal of Clinical Nursing, 28(9–10), 1794–1807. 10.1111/jocn.14789 30667129

[nop2719-bib-0030] Shao, H. , Yang, S. , Fonseca, V. , Stoecker, C. , & Shi, L. (2019). Estimating quality of life decrements due to diabetes complications in the United States: The Health Utility Index (HUI) diabetes complication equation. Pharmacoeconomics, 37(7), 921–929. 10.1007/s40273-019-00775-8 30778865 PMC7220804

[nop2719-bib-0031] Sirey, J. A. , Bruce, M. L. , Alexopoulos, G. S. , Perlick, D. A. , Friedman, S. J. , & Meyers, B. S. (2001). Stigma as a barrier to recovery: Perceived stigma and patient‐rated severity of illness as predictors of antidepressant drug adherence. Psychiatric Services, 52(12), 1615–1620. 10.1176/appi.ps.52.12.1615 11726752

[nop2719-bib-0032] Song, J. J. (2011). SPSS/AMOS statistics analysis method. : 21 Segisa.

[nop2719-bib-0033] Song, Y. , & Ah, E. (2016). Patients' perspectives on taking insulin in diabetes‐Perspectives of convergence. Journal of Digital Convergence, 14(12), 283–292. 10.14400/JDC.2016.14.12.283

[nop2719-bib-0034] Tabachnick, B. G. , Fidell, L. S. , & Osterlind, S. J. (2001). Using multivariate statistics. : Allyn and Bacon.

[nop2719-bib-0035] Vass, V. , Sitko, K. , West, S. , & Bentall, R. P. (2017). How stigma gets under the skin: The role of stigma, self‐stigma and self‐esteem in subjective recovery from psychosis. Psychosis, 9(3), 235–244. 10.1080/17522439.2017.1300184

[nop2719-bib-0036] Vogel, D. L. , Wade, N. G. , & Haake, S. (2006). Measuring the self‐stigma associated with seeking psychological help. Journal of Counseling Psychology, 53(3), 325–337. 10.1037/0022-0167.53.3.325

[nop2719-bib-0037] Woo, J. P. (2012). The understanding and concept of structural equation modeling. Han‐narae Academy.

